# Vaccination against SARS-CoV-2 provides low-level cross-protection against common cold coronaviruses in mouse and non-human primate animal models

**DOI:** 10.1128/jvi.01390-24

**Published:** 2025-01-16

**Authors:** Maedeh Naghibosadat, George Giorgi Babuadze, Yanlong Pei, Jacklyn Hurst, Elsa Salvant, Kayla Gaete, Mia Biondi, Badru Moloo, Alyssa Goldstein, Stacey Avery, Kathleen Ma, Anna Pietraszek, Sarah K. Wootton, Assad Alhaboub, Benjamin Martin, Samira Mubareka, Juan Corredor, Azmiri Sultana, Adebayo Adeekoa, Patrick Budylowski, Mario Ostrowski, Jesse Chao, Eva Nagy, Robert Kozak

**Affiliations:** 1 Sunnybrook Research Institute, Sunnybrook Health Sciences Centre 71545 , Toronto, Ontario, Canada; 2 University of Texas Medical Branch 12338 , Galveston, Texas, USA; 3 Department of Pathobiology, Ontario Veterinary College, University of Guelph 3653 , Guelph, Ontario, Canada; 4 School of Nursing, York University 7991 , Toronto, Ontario, Canada; 5 University Health Network 7989 , Toronto, Ontario, Canada; 6 Department of Laboratory Medicine and Pathobiology, University of Toronto 7938 , Toronto, Ontario, Canada; 7 St. Michael's Hospital, Unity Health 10071 , Toronto, Ontario, Canada; Universite Laval, Laval, Quebec, Canada

**Keywords:** coronavirus, vaccines, adaptive immunity, cross-protection

## Abstract

The common cold coronaviruses are a source of ongoing morbidity and mortality particularly among elderly and immunocompromised individuals. While cross-reactive immune responses against multiple coronaviruses have been described following severe acute respiratory syndrome coronavirus 2 (SARS-CoV-2) infection and vaccination, it remains unclear if these confer any degree of cross-protection against the common cold coronaviruses. A recombinant fowl adenovirus vaccine expressing the SARS-CoV-2 spike protein (FAdV-9-S19) was generated, and protection from SARS-CoV-2 challenge was shown in K18-hACE2 mice. Vaccinated mice were also challenged with the common cold coronaviruses human coronavirus (HCoV)-OC43 and HCoV-NL63 by the intranasal route, and viral shedding and lung burden were reduced in these groups compared to unvaccinated animals. Histopathological analysis of lung tissues revealed significantly less inflammation and lower pathology scores in mice that received FAdV-9-S19 . Because no mouse model for the coronavirus HCoV-229E exists, we vaccinated and challenged cynomolgus macaques to evaluate cross-protection against HCoV-229E. Animals were monitored for clinical signs of disease and viral shedding. Infectious virus was detected in both groups throughout the course of infection; however, vaccinated animals showed reduced viral shedding at multiple time points after infection. Histopathological analysis of lung tissues following challenge also indicated a more moderate disease in the vaccinated animals. Therefore, vaccination with FAdV-9-S19 also provided a moderate cross-protection against HCoV-229E disease in the cynomolgus macaques infection model. Our study demonstrates that vaccination with a recombinant fowl adenovirus expressing SARS-CoV-2 spike protein can provide a low-level cross-protection against beta- and alphacoronaviruses. These findings are important for the design of future pan-coronavirus vaccines.

**IMPORTANCE:**

The common cold coronaviruses are a source of ongoing morbidity and mortality particularly among elderly and immunocompromised individuals, and no vaccine is currently available. Cross-reactive immune responses have been described following severe acute respiratory syndrome coronavirus 2 (SARS-CoV-2) vaccination; however, it remains unclear what degree of cross-protection they confer against the common cold coronaviruses. We demonstrate that both humoral and cell-mediated immune responses provide a low-level cross-protection, resulting in reduced viral load and pathology for the common cold coronaviruses OC43 and NL63 in mouse models. Additionally, we present a novel non-human primate (NHP) model of infection with the common cold coronavirus 229E, demonstrating that it mimics the disease observed in humans and can serve as a model for future vaccine studies, as cross-protection was also observed. This is significant as it suggests that current vaccines could provide a low-level protection against other coronaviruses and could serve as part of vaccination strategy against future novel coronaviruses.

## INTRODUCTION

The coronavirus disease 2019 (COVID-19) pandemic has resulted in millions of deaths and hundreds of millions of cases worldwide. Vaccines have been an invaluable tool in reducing virus transmission, as well as COVID-19 severe disease and mortality. Current vaccines utilize the spike protein of severe acute respiratory syndrome coronavirus 2 (SARS-CoV-2) as the antigen, and it has been shown that the spike protein is a known B-cell and T-cell antigen, and common epitopes elicit a cross-reactive immune response (1). This is highlighted by multiple studies that have shown that various vaccine platforms can elicit broadly neutralizing antibodies against multiple sarbecoviruses (2). However, the degree of protection that vaccination confers against the human common cold coronaviruses remains less clear. These coronaviruses include members that are part of both the alphacoronaviruses (HCoV-229E, HCoV-NL63) and betacoronaviruses (HCoV-OC43, HCoV-HKU1). These are an annual cause of upper respiratory tract infections and result in hospitalization and mortality, particularly in immunocompromised individuals (3, 4).

There is conflicting evidence on the role that previous HCoV infection plays in protecting against SARS-CoV-2 (5–8). Several studies have shown that the cell-mediated immune response generated by SARS-CoV-2 infection and vaccination results in cross-reactivity to antigens from other alpha- and betacoronaviruses (9, 10). Recent work has highlighted that the T-cell immune response to HCoV antigens increased after COVID-19 exposure (9). Ng et al. showed cross-reactive antibodies to SARS-CoV-2 in serum samples collected prior to 2019 from individuals with HCoV infections (11). Additionally, they showed conserved core epitopes between the S2 region of SARS-CoV-2 spike protein and the glycoproteins of the seasonal CoVs. Greater homology was seen between SARS-CoV-2 and the betacoronaviruses compared to the alphacoronaviruses. Moreover, serological studies on pre-pandemic samples with previous HCoV exposure vary considerably in the percentage of samples with cross-reactive antibodies with numbers ranging from 14% to 90% (11, 12). Additionally, SARS-CoV-2 was shown to share common epitopes in the spike protein with HCoV-229E and HCoV-OC43, suggesting a potential for cross-protection (13).

Overall, it remains unclear if vaccination against SARS-CoV-2 provides cross-protection against the common cold coronaviruses (HCoVs). Because many individuals have been exposed to HCoVs and thus have background immunity, it is difficult to determine the level of cross-protection of SARS-CoV-2 vaccination using human samples. We sought to determine if cross-protection against HCoVs was generated following SARS-CoV-2 vaccination with an avian adenoviral vaccine. Additionally, as non-human primates (NHPs) have been shown to be good models of moderate disease for SARS-CoV-2, we investigated the pathogenesis and vaccine cross-protection in this model following an intranasal challenge with HCoV-229E.

## RESULTS

### Construction of two recombinant FAdV-9-S19 viruses

Plasmids containing the human codon optimized full-length S-gene from SARS-CoV-2 (Wuhan Hu-1) were introduced through recombination into the fowl-adenovirus-9 infectious clone, and virus was recovered (14). The presence of the S-gene and expression was confirmed by PCR and Western blot, respectively (Fig. S1). Virus titers were determined by plaque assay, and stocks were generated for animal vaccination experiments.

### Vaccine immunogenicity and protection against SARS-CoV-2

**Fig 1:**
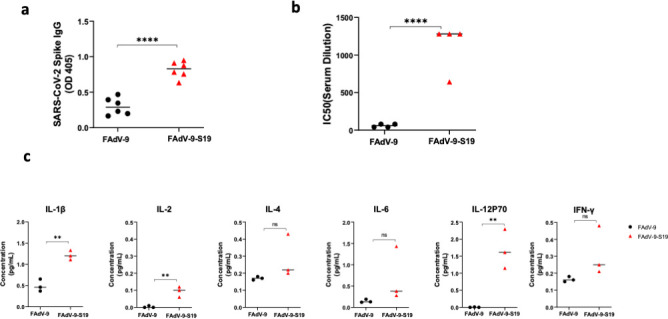
Humoral and cell-mediated immune responses in BALB/c mice. (a) Mice (*n* = 6 per group) were immunized with FAdV-9-S19 or empty vector, and Spike-specific antibodies from serum were detected by ELISA at 28 days post-vaccination. (b) Titers of neutralizing antibodies against SARS-CoV-2 in serum from mice that received FAdV-9-S19 or the empty vector (*n* = 4 per group). (c) Splenocytes from mice in each group (*n* = 3 per group) were pooled and stimulated with SARS-CoV-2 spike peptide pools (S1 and S2), and the cytokine concentrations were measured by a multiplex cytokine array. Error bars represent standard deviation, and the *t*-test was used for analysis. *P* value < 0.001 (**), *P* value < 0.0001 (****).

**Fig 2:**
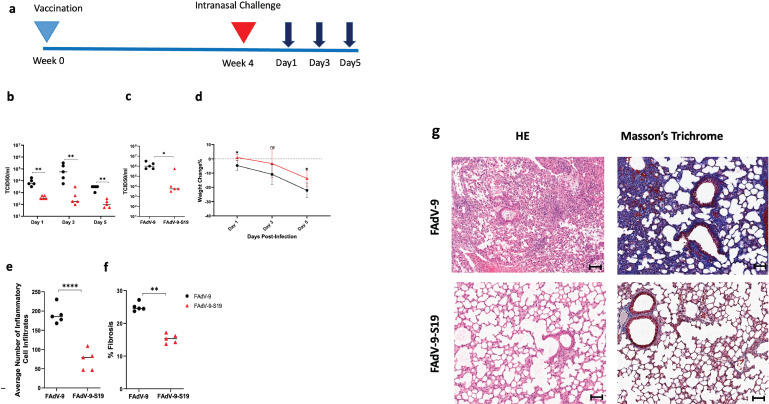
Vaccine protection in K18-hACE2 mice following SARS-CoV-2 challenge. (a) Experiment timeline with vaccination and challenge times denoted by blue and red triangles. Sampling timepoints are shown by the dark blue arrows. (b) SARS-CoV-2 viral loads from oral swabs and (c) lungs by TCID_50_. Error bars represent standard deviation. Significance was determined by two-way ANOVA with Dunnett’s multiple comparisons and *t*-test for oral swabs and lungs, respectively. *P* value < 0.001 (**), *P* value < 0.05 (*). (d) Animals were weighed throughout the course of infection, and percent weight change was compared to baseline weights (*n* = 5 per group). Error bars represent standard deviation. Two-way ANOVA was used for statistical analysis. *P* value < 0.01 (*). (e) Quantification of inflammatory cell infiltration in lung tissues (average number of inflammatory cell infiltrates per field of view in lung tissue). Error bars represent standard deviation. A *t*-test was performed for analysis. *P* value < 0.0001 (****) . (f) Quantification of fibrosis presented as mean percentage fibrosis of total lung tissue. Error bars represent standard deviation. A *t*-test was performed for analysis. *P* value < 0.001(**). (g) Representative hematoxylin and eosin (H&E) and Masson’s trichrome staining (20× magnification, scale bar = 100 μm) in vaccinated and sham-vaccinated mice.

Immunogenicity was evaluated in BALB/c mice, and groups were vaccinated with FAdV-9-S19 or the empty vector. No adverse reactions were observed. Serum was collected at 28 days post-vaccination (Fig. 1a). The total IgG titers against the SARS-CoV-2 spike protein were observed to be higher in animals that received the FAdV-9-S19 vaccine. Additionally, neutralizing antibody titers were examined and found to be higher in this group (Fig. 1b). To evaluate SARS-CoV-2-specific cell-mediated immunity, splenocytes were collected at 28 days post-vaccination and stimulated with peptides from the SARS-CoV-2 spike protein. As shown in Figure 1, higher Th1-cytokine levels were observed in the vaccinated mice. To demonstrate protection, vaccinated and sham-vaccinated hACE-2 mice were challenged with 3.16 × 10^6^ TCID50 (50% tissue culture infective dose) of SARS-CoV-2 (B1 lineage [15]) by the intranasal route. As shown in Figure 2, viral shedding was significantly reduced in mice that received FAdV-9-S19 compared to those that received the empty vector (Fig. 2b). Lung viral burden was also reduced in this group (Fig. 2c). Both groups showed weight loss following infection, but it was greater in the empty vector group, although this failed to achieve statistical significance (Fig. 2d). Histopathological analysis of the lungs was performed following euthanasia of the animals, and the extent of pulmonary disease was evaluated. There was significantly less inflammatory cell infiltrates and fibrosis and lower lung pathology scores in mice that received FAdV-9-S19 (Fig. 2e through g) . These data indicated that vaccination with FAdV-9-S19 confers protection against SARS-CoV-2 by reduced viral shedding, lung burden, and pathology.

### Vaccination confers low-level cross-protection against HCoV-NL63 and HCoV-OC43

**Fig 3:**
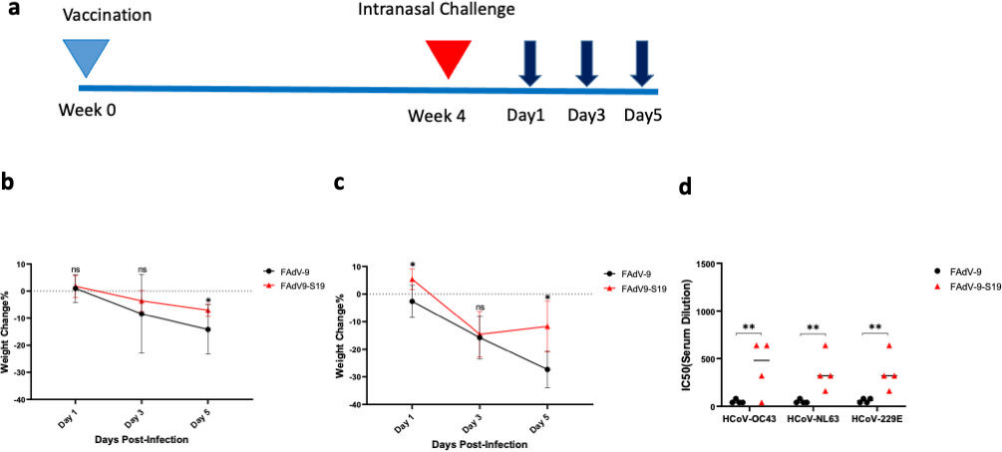
Protection in hACE-2 K-18 mice following challenge with HCoV-OC43 or HCoV-NL63. (a) Vaccination and challenge schedule. Sampling timepoints are shown by the dark blue arrows. Mice were weighed daily following infection with either (b) HCoV-OC43 or (c) HCoV-NL63. Data represent the percent weight change compared to pre-infection weights (*n* = 5 per group). Data points represent mean percent weight changes; error bars represent standard deviation. Two-way ANOVA was used for statistical analyses. *P* value < 0.05 (*). (d) Serum from mice from each group was analyzed for neutralization against HCoV-OC43, HCoV-NL63, and HCoV-229E (*n* = 4 per group). Two-way ANOVA with repeated measures was used for analysis. *P* value < 0.001 (**).

**Fig 4:**
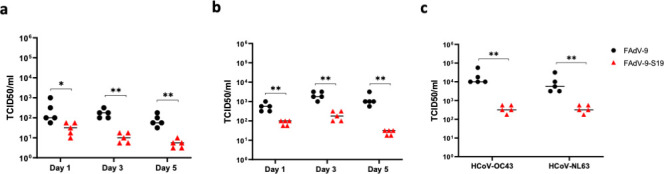
HCoV-OC43 and HCoV-NL63 viral shedding in vaccinated and sham-vaccinated mice. Titers of infectious virus were determined from oral swabs of hACE-2 K-18 mice challenged with (a) HCoV-OC43 and (b) HCoV-NL63. Error bars represent standard deviation. (c) Viral titers in lung tissues at 5 days post-infection with HCoV-OC43 or HCoV-NL63. Two-way ANOVA using Dunnett’s multiple comparisons was used for analysis. *P* value < 0.001 (**), *P* value < 0.05 (*).

**Fig 5:**
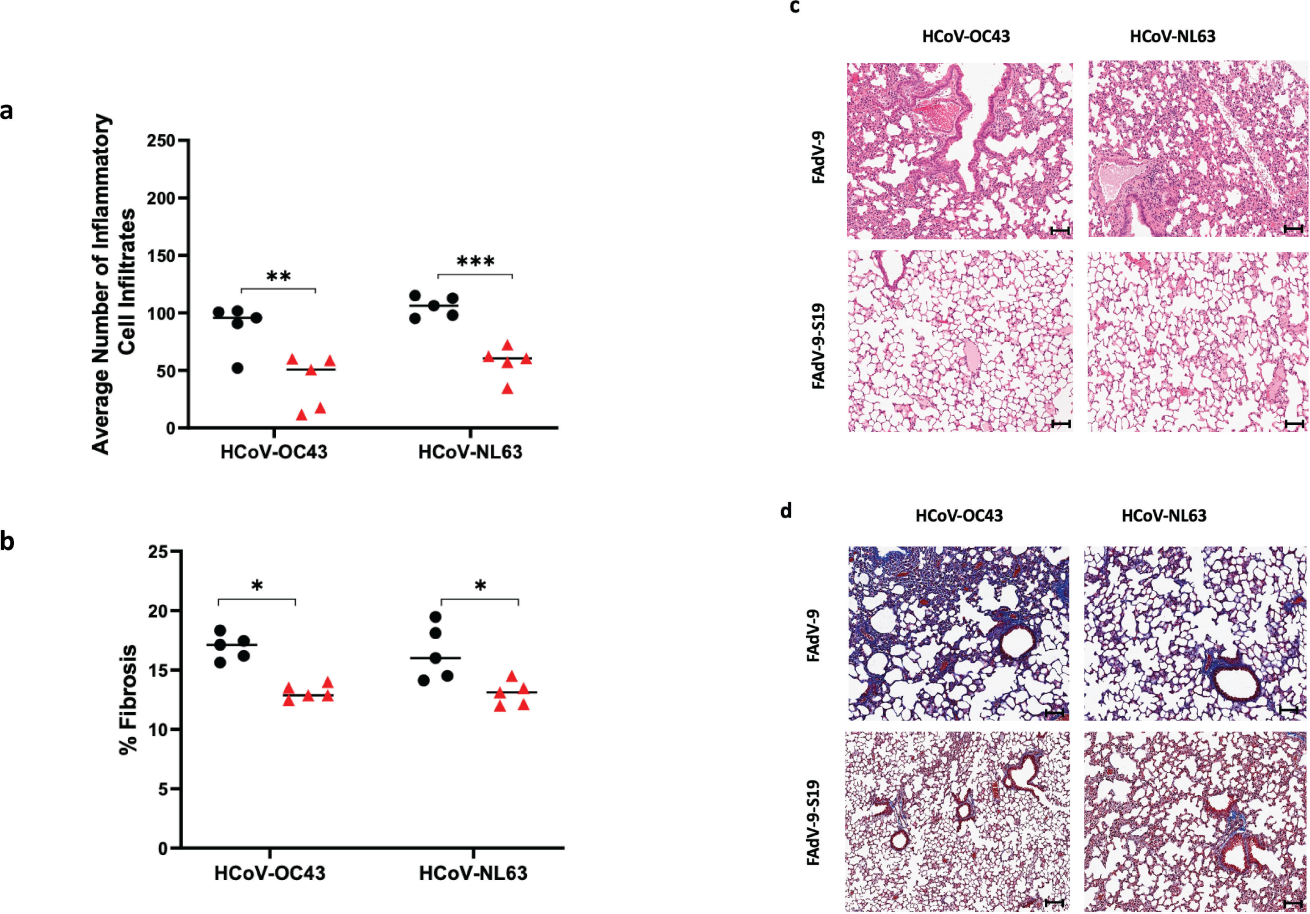
Lung pathology in vaccinated and unvaccinated hACE-2 K-18 mice challenged by HCoV-OC43 and HCoV-NL63. (a) Quantification of inflammatory cell infiltration in lung tissues (average number of inflammatory cell infiltrates per field of view in lung tissue). Two-way ANOVA with Dunnett’s multiple comparisons test was performed (FAdV-9 vs FAdV-9-S19 [HCoV-OC43 challenge], ***P < 0.0001; FAdV-9 vs FAdV-9-S19 [HCoV-NL63 challenge], **P < 0.001). Error bars represent standard deviation. (b) Quantification of fibrosis presented as mean percentage fibrosis of total lung tissue. Two-way ANOVA with Dunnett’s multiple comparisons test was performed (FAdV-9 vs FAdV-9-S19 [HCoV-OC43 challenge], *P < 0.05; FAdV-9 vs FAdV-9-S19 [HCoV-NL63 challenge], *P < 0.05). Error bars represent standard deviation. (c) Lung histopathology and (d) fibrosis in vaccinated and sham-vaccinated mice challenged by HCoV-OC43 or HCoV-NL63 (*n* = 5 per group). Images are 20× magnification, scale bar = 100 μm.

Sequence alignment of the spike protein of SARS-CoV-2 and seasonal coronaviruses has shown moderate amino acid homology and common epitopes (16), suggesting the potential for cross-protection. To investigate this, we first examined antibody cross-reactivity. We performed virus neutralization assays to measure the titers of neutralizing antibodies (nAbs) in the serum of BALB/c mice (collected at 28 days post-vaccination) against HCoV-NL63, HCoV-OC43, and HCoV-229E. For all HCoVs, nAb titers were greater in the vaccinated mice (Fig. 3d), but levels were lower compared to the nAb titers against SARS-CoV-2 (Fig. 1b). Next, we evaluated cross-protection against severe disease in hACE-2 mice challenged intranasally with HCoV-OC43 (1.78 × 10^6^ TCID_50_) or HCoV-NL63 (3.16 × 10^6^ TCID_50_) (Fig. 3). Infection with either HCoV-OC43 or HCoV-NL63 resulted in weight loss that was more pronounced in the group that received the empty vector, yet not statistically significant (Fig. 3b and c). Viral shedding was measured from oral swabs at 1, 3, and 5 dpi. Viral loads were significantly lower across all time points in oral swabs collected from vaccinated mice compared to unvaccinated mice following infection with either HCoV-OC43 or HCoV-NL63 (Fig. 4a and b). At 5 dpi, mice were euthanized, and viral burden in the lungs was examined. K18-hACE2 mice vaccinated with FAdV-9-S19 showed significantly lower infectious viral loads of both seasonal coronaviruses (Fig. 4c), compared to mice that received only the empty vector. The viral RNA levels in lungs and oral swabs were also lower in the vaccinated animals (Fig. S4). Additionally, examination of lung pathology also revealed reduced inflammation and fibrosis in the vaccinated groups (Fig. 5a through c). Overall, vaccinated mice were better protected against lower respiratory tract pathology from infection with HCoV-OC43 and HCoV-NL63 compared to unvaccinated animals.

### Vaccination confers low-level cross-protection against HCoV-229E in non-human primates

**Fig 6:**
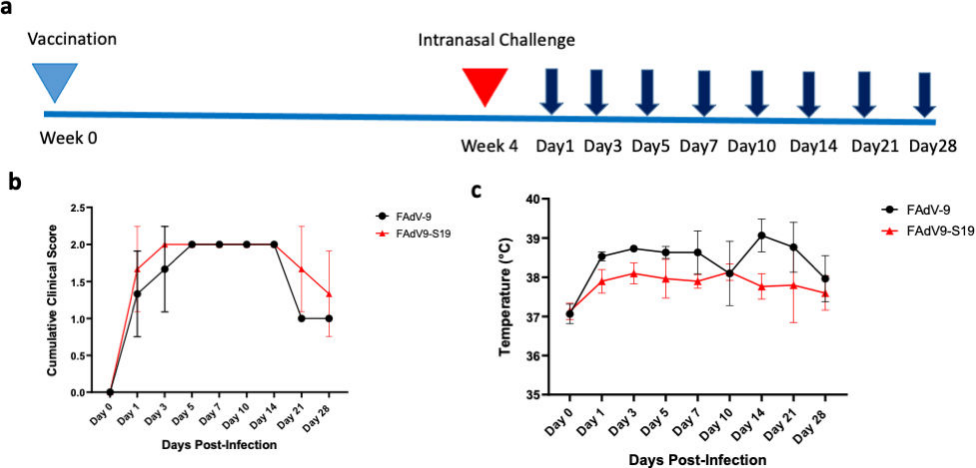
Non-human primates clinical scores and body temperatures following infection with HCoV-229E. (a) Vaccination and challenge schedule. Sampling timepoints are shown by the dark blue arrows. Animals in both groups were monitored for (b) fever and (c) clinical scores over the course of infection (*n* = 3 per group). Two-way ANOVA was used for analysis.

Because there is no mouse model for HCoV-229E infection, we vaccinated and challenged cynomolgus macaques to evaluate cross-protection. Animals received an intramuscular (IM) vaccination of FAdV-9-S19 or empty vector and were monitored for 4 weeks (Fig. 6). In both groups, vaccinations were well tolerated, and no adverse events were observed. Next, animals were given a nasal challenge with 1.78 × 10^6^ TCID_50_ of HCoV-229E virus and were monitored for clinical signs of disease and viral shedding. Animals developed characteristic signs of an upper respiratory tract infection (Fig. 6; Table 1) including elevated body temperatures. Hematological and biochemical markers showed a decrease in white blood cell counts following infection, when compared to baseline (Fig. S2) in both groups. The disease presentation was similar in both vaccinated and empty-vector groups. Animals in both groups developed a fever and had increased clinical scores over the course of infection (Fig. 6; Table 1). While temperatures were moderately lower in the vaccinated group, differences did not reach statistical significance. Blood biochemistry was comparable between both groups. Interestingly, animals that received FAdV-9-S19 showed mild lymphopenia at 10 dpi, although this was not statistically significant (Fig. S1). Chest X-rays also indicated mild infection in both groups (data not shown).

**TABLE 1:** Clinical findings in non-human primates challenged with HCoV-229E

Animal ID	Vaccine	Species	Sex	Age	Weight (kg)	Route of infection	Fever	Symptoms
FR1016	FAdV-S19	Cynomolgus	M	3 years	4.69	Intranasal	Fever on days 1, 3, 5 post-infection	Urination and defecation on days 3 and 5 post-infection
FR486A	Vector only	Cynomolgus	M	3 years	4.29	Intranasal	Fever on days 1, 3, 5, 7, 10, 14, 21, 28 post-infection	Urination and defecation on days 1, 3, 5, 7, 10, 14 post-infection. Mild crusting, nasal discharge, soft stool
FR981	Vector only	Cynomolgus	M	3 years	4.06	Intranasal	Fever on days 1, 3, 5, 7, 10, 14, 21, 28 post-infection	Urination and defecation on days 1, 3, 5, 7, 10, 14, 21 post-infection. Small amount of blood in right nostril. Fecal staining on day 14 post-infection. Wet stool around anus on day 7 post-infection. Mild crusting, nasal discharge. Tracheobronchial lymph node enlargement. Alopecia on left hip and along the tail
MB450	FAdV-S19	Cynomolgus	M	3 years	4.26	Intranasal	Fever on days 1, 3, 5, 7 post-infection	Urination and defecation on days 1, 3, 5 post-infection
MB672	FAV-S19	Cynomolgus	M	3 years	4.78	Intranasal	Fever on days 1, 3, 5 post-infection	Urination and defecation on days 5 and 7 post-infection. Mild alopecia along the tail
UG866	Vector only	Cynomolgus	M	3 years	4.04	Intranasal	Fever on days 1, 3, 5, 7, 10, 14, 21, 28 post-infection	Urination and defecation on days 1, 3, 5, 7, 10, 14 post-infection. Mild crusting, nasal discharge. Alopecia along the tail

### Vaccination reduces viral shedding and pathology

**Fig 7:**
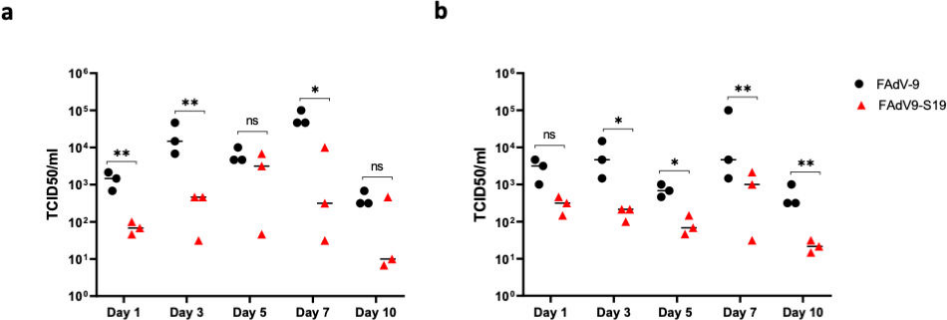
Viral shedding in HCoV-229E-challenged non-human primates. Animals (*n* = 3 per group) were challenged with HCoV-229E and monitored for viral shedding. Titers of infectious virus from various time-points were collected and determined by TCID_50_ from (a) nasal swabs and (b) oral swabs. Error bars represent standard deviation. Two-way ANOVA with repeated measures was used for statistical analysis. *P* value < 0.001 (**), *P* value < 0.05 (*).

**Fig 8:**
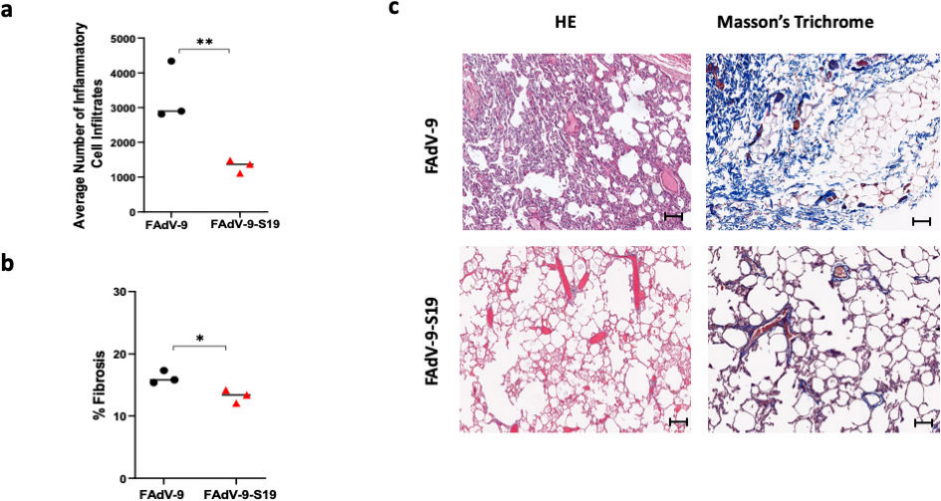
Lung and spleen pathology in non-human primates challenged by HCoV-229E. (a) Quantification of inflammatory cell infiltration in lung (average number of inflammatory cell infiltrates per field of view in lung tissue). (b) Quantification of fibrosis presented as mean percentage fibrosis of total lung tissue. Lung histopathology and (c) fibrosis in vaccinated and sham-vaccinated animals challenged by HCoV-229E (*n* = 3 per group). Images are 20× magnification, scale bar = 100 μm. Error bars on graphs represent standard deviation. A *t*-test was performed for analysis. *P* value < 0.001 (**), *P* value < 0.05 (*).

**Fig 9:**
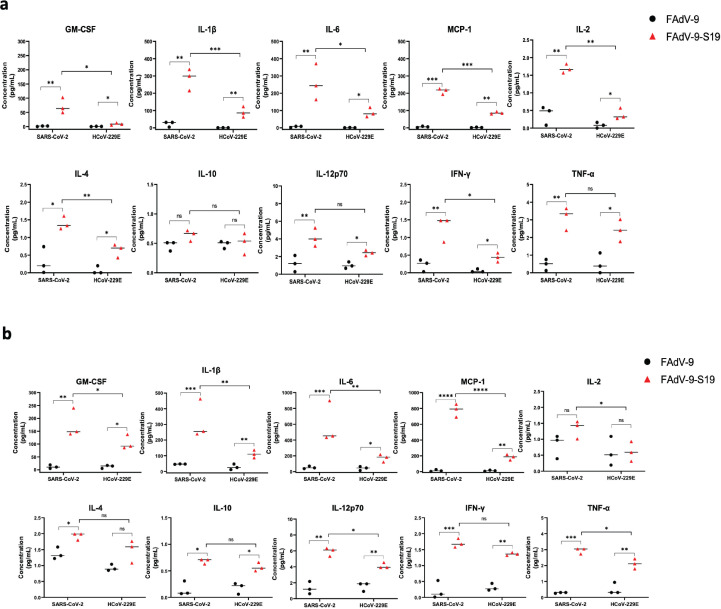
Cell-mediated responses in vaccinated and sham-vaccinated non-human primates. PBMCs from animals in each group (*n* = 3 per group) were pooled and stimulated with SARS-CoV-2 spike peptide pools (S1 and S2) and HCoV-229E spike peptide pool. Cytokine concentrations were measured by a multiplex cytokine array at (a) 15 days post-vaccination and (b) 5 days post-infection. Error bars represent standard deviation. Two-way ANOVA was used for analysis. *P* value < 0.0001 (***), *P* value < 0.001 (**), and *P* value < 0.05 (*).

Oral and nasal swabs were collected to monitor viral shedding over the course of infection. Infectious virus was detected in both vaccinated and unvaccinated groups throughout the course of infection; however, vaccinated animals showed reduced viral shedding following infection (Fig. 7). Because vaccination with FAdV-9-S19 reduced lung pathology in mice following SARS-CoV-2 infection, we investigated if similar findings were seen in NHPs following HCoV-229E challenge. Four weeks after infection, the primates were euthanized, and lungs were analyzed for evidence of inflammation and fibrosis using an automated pipeline for image analysis (Fig. 8a through c). Inflammatory scores were similar for animals in both groups, but fibrosis scores were lower in the vaccinated animals. To examine if cross-reactive cell-mediated immunity may have been contributing to the observed protection, we collected peripheral blood mononuclear cells (PBMCs) following vaccination, and after intranasal challenge, stimulated them with peptides from the spike proteins of SARS-CoV-2 and HCoV-229E. As shown in Figure 9, higher levels of Th1 cytokines were observed in vaccinated compared to unvaccinated animals when stimulated with spike peptides from either coronavirus. However, greater cytokine production was seen in PBMCs from vaccinated animals stimulated with SARS-CoV-2 spike peptides. Collectively, these data indicate that vaccination with FAdV-S19 provided a low-level cross-protection against HCoV-229E challenge.

## DISCUSSION

In this study, we demonstrate that immunization with a vaccine expressing the SARS-CoV-2 spike protein provided a low-level cross-protection against common cold coronaviruses, including reduction in viral shedding, lung burden, and pathology, in mice following intranasal challenge. We also show that the NHP model of HCoV-229E infection mimics aspects of disease observed in humans, as animals developed characteristic signs of an upper respiratory tract infection, including elevated body temperature, mild nasal discharge, and tracheobronchial lymph node enlargement (3, 17). Hematological and biochemical markers showed a decrease in white blood cell counts following infection. Interestingly, we noted an inflammation response in the NHP lungs following necropsy, suggestive of immunopathology (18). This is similar to what has been reported in human infections, as it was noted in a multi-year study that dyspnea was more frequently associated with HCoV-229E infection (19). Moreover, the group of animals that received the adenoviral-based SARS-CoV-2 vaccine and subsequent challenge with HCoV-229E also showed a low-level cross-protection. Recently, hamster studies where animals were challenged first with HCoV-OC43, allowed to recover, and subsequently challenged with SARS-CoV-2 showed faster viral clearance (20). Notably, our study demonstrated that vaccination conferred broader protection and more disease moderation compared to natural infection, although differences in animal models must be taken into consideration.

The cross-protection against common cold coronaviruses observed in our animal studies is likely due to a combination of humoral and cell-mediated immune responses, as we observed both cross-reactive nAbs and production of Th1 cytokines in PBMCs in response to spike peptides from HCoV-229E. This is similar to what has been reported in several studies examining vaccination in humans. Hu et al. (21) noted that individuals who received an inactivated SARS-CoV-2 vaccine also had elevated titers of antibodies against HCoV-OC43 compared to pre-vaccination, supporting the idea that broader coronavirus immunity can be generated (21).

Furthermore, HCoV-OC43 receptor-binding domain (RBD)-specific IgM and IgG1 were expanded in survivors of severe disease (22). This is further supported by the work of Cantoni et al., who demonstrated that mRNA vaccination (with mRNA-1273) increased neutralizing antibodies against HCoV-NL63 and that vaccination with AZD1222 (AstraZeneca vaccine) increased HCoV-229E neutralizing antibody titers (23). Similar studies examining neutralizing antibody titers against common cold coronaviruses showed a fourfold increase in antibodies against HCoV-229E following SARS-CoV-2 vaccination, despite the low level of sequence homology of spike proteins (24). The S2 region of SARS-CoV-2 spike protein contains conserved regions that are targeted by antibodies and have been found in serum samples collected prior to the COVID-19 pandemic, suggesting that epitopes in this region may be broadly protective (11). Reduced humoral immunity is associated with COVID-19 mortality, and antibody responses against S2 predominate in individuals with severe disease (22). Moreover, binding antibodies have been associated with protection against severe COVID-19 (25), and a similar mechanism may occur in the case of the common cold coronaviruses, whereby the immune response may not prevent infection but limits disease severity. This is similar to what was observed in our mouse challenge experiments, where viral loads were reduced, but still present, in the lungs of vaccinated mice compared to unvaccinated animals, suggesting cross-protection. In related studies, it has been shown that mice immunized with the RBDs from SARS-CoV-1, SARS-CoV-2, or the bat CoV, RaTG13, generated cross-neutralizing antibodies against a common epitope found in multiple sarbecoviruses (26). Similar studies carried out with non-human primates also showed that antibodies generated from vaccination were effective at neutralizing multiple SARS-CoV-2 variants of concern as well as bat sarbecoviruses (27).

Cell-mediated immunity is essential to preventing severe disease in coronavirus infections, and sequence alignment of the spike protein of SARS-CoV-2 and the common cold coronavirus has shown moderate amino acid homology and potential common epitopes (16), suggesting the potential for cross-protection. Our data support this, as moderate levels of Th1 cytokines were produced in PBMCs stimulated with HCoV-229E peptides. Future studies will look at the potential for cross-reactivity with peptides from other coronavirus spike proteins. Immunological investigations in health-care workers demonstrated that T-cells that are cross-reactive for SARS-CoV-2 and seasonal coronaviruses may have a mild protective effect by rapidly controlling SARS-CoV-2 infection (28). Additionally, higher frequencies of CD4 and CD8 T-cells that were cross-reactive with epitopes from the common cold coronaviruses were reported in unvaccinated COVID-19 patients with mild disease compared to those with more severe diseases, suggesting previous exposures may aid in providing protection (29). This aligns with our findings whereby NHPs in our vaccinated group had lower viral burden in the lungs and reduced evidence of fibrosis compared to the unvaccinated group, likely due to cross-protective T-cells. Notably, it has been observed that T-cells had higher reactivity against the S1 antigen of HCoV-OC43 and HCoV-NL63, and against the S2 antigen of HCoV-OC43 and HCoV-229E in COVID-19 survivors (9). Additionally, studies in animals and humans have demonstrated that vaccination with the spike antigen from the original Wuhan isolate can provide cross-protection against the SARS-CoV-2 variants of concern, likely a result of common epitopes (30–32). Moreover, patients with agammaglobulinemia and other B-cell deficiencies highlight the role played by T-cells in protection, as these patients developed pneumonia but did not require oxygen or admission to the ICU, in the case of SARS-CoV-2 infection (33).

Studies have shown that a greater immune response against SARS-CoV-2 variants is observed when multiple vaccine doses are given, and determining whether this also increases cross-protective immunity to other alpha- and betacoronaviruses should be a goal of future studies.

Moreover, cross-protection induced by other coronavirus antigens remains to be evaluated. Data from health-care workers in the Netherlands found an association between antibodies against the nucleocapsid of HCoV-OC43 and protection (8). Recent studies have identified numerous coronaviruses exist in bats and have potential for spillover, thereby increasing the risk of subsequent pandemics (34, 35). Evaluating the degree of cross-protection conferred by current vaccines could help determine their potential as a stop-gap measure until more tailored vaccines are designed and could serve as the basis for future heterologous vaccination strategies. Additionally, the common cold human coronaviruses are an annual cause of hospitalization and mortality, particularly in immunocompromised individuals (3, 4). Thus, clinical studies to determine if current SARS-CoV-2 vaccines confer low-level protection should be investigated. In conclusion, our study demonstrates the potential for vaccination to induce a moderate cross-protection against alpha- and betacoronaviruses. Future studies investigating how the route of vaccination and the addition of other coronavirus antigens improves cross-protection, with the goal of developing a pan-coronavirus vaccine, should be pursued.

## MATERIALS AND METHODS

### Construction of recombinant vaccines

The FAdV-9 strain A-2A (ATCC VR-833) virus and its derivatives were propagated in CH-SAH cells. The cells were maintained in Dulbecco’s Modified Eagle’s Medium (DMEM)/F12 supplemented with 10% fetal bovine serum (FBS), 2 mM L-glutamine, 100 U/mL penicillin, and 100 µg/mL streptomycin. Virus titers were determined by plaque assay, and all viruses were used at a multiplicity of infection (MOI) of 5 for cell infections. The pFAdV-9-S19-SwaI backbone was used to generate two recombinant pFAdV-9 fadmids (infectious clone consisting of the viral genome cloned into a cosmid vector) following the procedures described previously (14, 36, 37). Briefly, the S gene was amplified through PCR using the forward primer Covid-S-NheI-F and the reverse primer Covid-S-NotI-R (Covid-S-NheI-F: AGCTGCTAGCCACCATGTTTGTTTTTCTTGTTTTATTGCC; Covid-S-NotI-R: AGCTGCGGCCGCTTAGGTATAATGCAACTTGAC). The amplified S gene was then used to replace GFP in the pEGFP-N1 plasmid, resulting in the creation of pEGFP-S. Next, the expression cassette (consisting of CMV promoter-S gene-PolyA, CA) was amplified using CA-SwaI-F and CA-SwaI-R primers (CA-SwaI-F: AGCTGCATTTAAATGTATTACCGCCATGCATTAG; CA-SwaI-R: AGCTGCATTTAAATCGCCTTAAGATACATTGATGAG). The amplified cassette was digested with SwaI, purified from the gel, and ligated to pFAdV-9Δ19-SwaI to generate the pFAdV-9Δ19-CA-CovidS construct. To generate pFAdV-9Δ19-NP-CovidS (native promoter, NP), the S gene was amplified using Covid-S-SwaI-F and Covid-SwaI-R primers (Covid-S-SwaI-R: AGCTGCATTTAAATTTATGTGTAATGTAATTTGACTCC; Covid-S-SwaI-F: AGCTGCATTTAAATATGTTTGTTTTTCTTGTTTTATTGCC). The amplified S gene was then digested with SwaI, purified, and inserted into the SwaI site of pFAdV-9-S19-SwaI plasmid.

The resulting FAdmid constructs were verified through NotI digestion and PCR amplification using the Ver-ORF19-F and Ver-ORF19-R primer set (Ver-ORF19-F: CAACTGACTACGGAATACAGGG; Ver-ORF19-R: CTAGTTTGCTTCCGCGTACG) and sequencing the insert and junction region. Two micrograms of each verified FAdmid was digested with PacI to linearize the DNA and then transfected into CH-SAH cells using Lipofectamine (Life Technologies, Canada). Two recombinant FAdV-9-S19 viruses were rescued 7 days post-transfection. Western blot analysis of whole cell lysates from CH-SAH cells infected with recombinant viruses was performed to confirm S protein expression. On post-infection day 3, the cells were lysed in RIPA buffer containing a protease inhibitor mix. Equal amounts of protein (50 µg) were separated by SDS-PAGE on a 12% gel and were transferred onto a polyvinylidene fluoride (PVDF) membrane. The membranes were then probed overnight at 4°C with rabbit primary antibodies against the SARS-CoV-2 spike protein (1:1,000 dilutions). After washing, the membranes were incubated with goat anti-rabbit horseradish peroxidase (HRP)-conjugated secondary antibodies (1:5,000 dilutions) for 1 hour at room temperature. Protein bands were visualized using an enhanced chemiluminescence (ECL) substrate and were imaged using a ChemiDoc imaging system.

### Recombinant FAdV-9 virus preparation

Three viruses, two rFAdV-9-S19 and FAdV-9 (empty vector), were grown using CH-SAH cells as has been previously described (14, 36). Briefly, CH-SAH cells at 90% confluence were infected with each virus at an MOI of 5 and were harvested on post-infection day 3. The harvested cells were frozen and thawed three times, followed by centrifugation at 2,200 × *g* for 10 minutes to remove cell debris. The resulting supernatant, along with a 30% sucrose cushion, was centrifuged again at 54,000 × *g* for 2 hours. The supernatant was discarded, and the pellet was resuspended in phosphate-buffered saline (PBS). A gradient was created in a 14 mL Beckman tube by slowly layering 3.5 mL of 1.35 g/mL CsCl, followed by 3 mL of 1.25 g/mL CsCl. The virus supernatant was carefully added to the tube and centrifuged at 54,000 × *g* for 1.5 hours. The resulting band was isolated using an 18 G × 1 ½" needle and was resuspended in a total of 8 mL of PBS. The virus was then dialyzed using a 10 K molecular-weight cutoff (MWCO) dialysis cassette.

### Mouse vaccination, challenge, and viral load quantification

Four-to-six-week-old female BALB/c and B6.Cg-Tg (K18-hACE2)2Prlmn/J mice were purchased from Charles River and The Jackson Laboratory, respectively. Animals were housed at the BSL2 facility at the University of Toronto and were transferred to the BSL3 facility at the University of Toronto for challenge experiments. For immunogenicity experiments, BALB/c mice were injected with 50 µL per caudal thigh of either Fowl Adenovirus 9 expressing S-antigen from SARS-CoV-2 (FAdV-9-S19) or Fowl Adenovirus 9 (FAdV-9) only as a vehicle control diluted in PBS (Gibco, Canada). A total of 100 µL (1 × 10^8^ PFU) was administered to each animal as a single dose. K18-hACE2 mice were challenged intranasally with 50 µL of either SARS-CoV-2 (B1 lineage, dose 3.16 × 10^6^ TCID50), HCoV-OC43 (BEI Resources, VR-1558; dose 1.78 × 10^6^ TCID50), or HCoV-NL63 (ATCC, Cat#: NR-470; dose 3.16 × 10^6^ TCID50) 4 weeks after vaccination. The viruses used for the animal experiments were passage 4 of SARS-CoV-2 (B1 lineage), passage 5 of HCoV-OC43, and passage 4 of HCoV-NL63, which were cultured in Vero E6, HCT-8, and LLC-MK2 cells, respectively. Vero E6 and HCT-8 cells were cultured in DMEM containing 2% FBS, 100 IU/mL penicillin, and 100 µg/mL streptomycin (Gibco, Canada). LLC-MK2 cells were cultured in Eagle's minimum essential medium (EMEM) containing 2% FBS, 100 IU/mL penicillin, and 100 µg/mL streptomycin (Gibco, Canada). Oral swabs were collected on 1, 3, and 5 days post-infection (dpi) in DMEM, while lung tissues were collected following the euthanasia of animals at 5 days post-infection. Quantities of infectious virus were determined by adding liquid from the collected swabs or homogenized tissues to Vero E6 for SARS-CoV-2, HCT8 for HCoV-OC43, and LLC-MK2 cells for HCoV-NL63 (38), respectively. Prior to infection, cells were seeded into 96-well plates and incubated overnight at 37°C. On the following day, media were aspirated, and samples were added and serially diluted (10-fold dilution) into subsequent wells followed by 1 hour of incubation at 37°C or 34°C with shaking every 10–15 minutes. After incubation, media were aspirated and replaced with complete DMEM or EMEM, and cells were again incubated at 37°C or 34°C and assessed for cytopathic effect (CPE) after 5, 7, and 10 days depending on the virus (31, 38, 39). The TCID50 was determined using the Spearman-Karber method.

### RT-qPCR

The viral RNA loads from oral swabs and homogenized tissues were extracted using the QIAamp viral RNA kit (QIAGEN, Canada) according to the manufacturer’s instructions. Detection and quantification of the HCoV-OC43 and HCoV-NL63 viral RNA were performed using the Luna Universal Probe One-Step RT-qPCR kit (New England Biolabs, Canada) as described previously by Peci and colleagues (40). For quantification, standard curves were generated using dilutions of HCoV-OC43 and HCoV-NL63 viruses.

### Cytokine analysis from mouse splenocytes

Cytokine concentrations from stimulated mouse splenocyte supernatants were measured. Splenocytes were collected, pooled within treatment groups, and added onto 96-well plates at 1 × 10^5^ splenocytes per well. Pooled splenocytes were stimulated with peptide pools (JPT Peptide Technologies GmbH, Berlin, Germany) containing 158 peptides derived from SARS-CoV-2 and spanning the complete spike protein. The peptide pools were applied at a final concentration of 1 µg/mL. The same volume of 40% dimethyl sulfoxide (DMSO) (Sigma-Aldrich), the solution to dissolve each peptide pool, was used as the negative control. PMA/Ionomycin (Sigma-Aldrich) was used as the positive control. After 18 hours of incubation at 37°C with 5% CO_2_, splenocyte supernatants were removed for cytokine analysis. Cytokine concentrations from unvaccinated mouse spleens were measured for background control. A “Mouse Cytokine Pro-inflammatory Focused 10-plex Discovery Assay (MDF10)” was performed by Eve Technologies (Calgary, AB, Canada) using the Luminex 200 system (Luminex, Austin, TX, USA) to simultaneously measure the concentrations of IL-1β, IL-2, IL-4, IL-6, IL-12p70, MCP-1, and TNF-α cytokines from three replicates per treatment group.

### Cytokine analysis—peripheral blood mononuclear cell (PBMC)

NHP PBMCs (*n* = 3 per group) from 15 days post-vaccination and 5 days post-infection were retrieved, thawed, and washed three times with RPMI, PBS, and S media (RPMI supplemented with 10% FBS, 1% non-essential amino acids, 1% Glutamine-Penicillin-Streptomycin, 0.1% beta*-*mercaptoethanol). A total of 250,000 cells were plated per well for T-cell stimulation.

PBMCs were stimulated with either SARS-CoV-2 or HCoV-229E peptide pools (JPT Peptide Technologies GmbH, Berlin, Germany). The SARS-CoV-2 peptide pool contains 158 peptides derived from SARS-CoV-2 and spanning the complete spike protein. HCoV-229E peptide pool contains 291 peptides derived from HCoV-229E and spanning the complete spike glycoprotein. Each peptide stock was resuspended in 50 µL DMSO to make a stock concentration of 500 µg/mL. Peptide stocks were diluted to a workable concentration of 1 µg/mL with a calculated amount of S media. T-cell stimulation involved four conditions: SARS-CoV-2 S peptide, HCoV-229E, PMA/ionomycin (positive control), and DMSO (negative control). Each well was plated with 100 µL of cells, and each condition was pipetted into its corresponding well with 100 µL, for a total volume of 200 µL per well. The plated cells were incubated at 37℃ with 5% CO_2_ for 48 hours. PMA/ionomycin (50 µL) was plated 5 hours prior to completing the 48 hour incubation. At the 48 hour mark, cytokine supernatant collection was performed where 75 µL of sample was placed in vials and sent to Eve Technologies for cytokine analysis. The multiplexing analysis was performed using the Luminex 200 system (Luminex, Austin, TX, USA) by Eve Technologies Corp. (Calgary, AB, Canada). Ten markers were simultaneously measured in the samples using Eve Technologies’ Non-Human Primate Cytokine Panel A 10-Plex Custom Assay (MilliporeSigma, Burlington, MA, USA) according to the manufacturer’s protocol. The 10-plex consisted of GM-CSF, IFN-γ, IL-1β, IL-2, IL-4, IL-6, IL-10, IL-12(p70), MCP-1, and TNF-α. Cytokine concentrations from DMSO (negative control) were measured for background control and subtracted from the actual cytokine concentration.

### Enzyme-linked immunosorbent assay (ELISA) for SARS-CoV-2 antibodies

Blood was obtained from mice via the lateral saphenous vein 28 days after vaccination, and serum was separated by centrifugation. Plates were coated with 20 µg per plate of NR-722, a truncated and glycosylated recombinant form of the SARS-CoV-2 spike glycoprotein (BEI Resources, Manassas, USA) diluted in 1× PBS and incubated overnight at 4°C. Plates were then washed with 1× PBS containing 0.05% Tween 20 and blocked with KPL Milk-Blocking Solution. After blocking, plates were washed and mice sera with the dilution of 1:400 were added. Plates were incubated at 37°C for 90 minutes, washed, and then incubated with anti-mouse-HRP secondary antibody at a 1:2,000 dilution. Following washing, the substrate was added as per manufacturer instructions (KPL two-component ABTS substrate). Absorbance at the 450 nm wavelength was determined. Sera from naive mice were used as an internal control on each assay group. A plate cutoff value was determined based on the average absorbance of the naive control starting dilution plus two standard deviations. Only sample dilutions whose average was above this cutoff were registered as a positive signal.

### Virus neutralization assays

Titers of neutralizing antibodies from BALB/c mouse sera were determined using the methods described by Abe and colleagues (41). Mice sera were diluted twofold from 1:20 to 1:2,560 in DMEM or EMEM supplemented with 1% penicillin/streptomycin and were incubated with 400 TCID50 of the virus (SARS-CoV-2, HCoV-OC4, or HCoV-NL63) at 37°C and 5% CO_2_ for 1 hour. After incubation, these mixtures were added to 96-well plates containing confluent Vero E6, HCT-8, and LLC-MK2 cells, and were incubated at 37°C or 34°C with 5% CO_2_ for 1 hour (31, 38, 39). After this, the media were aspirated and replaced with DMEM or EMEM containing 2% FBS and 1% penicillin/streptomycin. Plates were assessed for CPE after 5, 7, and 10 days, and virus neutralization titers were recorded as the reciprocal of the highest dilution of serum where the CPE was recorded.

### Histopathology of mouse organs

Tissues were fixed in 10% neutral phosphate-buffered formalin, routinely processed, sectioned at 5 µm, and stained with hematoxylin and eosin (H&E) for histopathologic examination. Lung tissues were examined for the presence or absence of features of cell or tissue damage (necrosis of bronchiolar epithelial cells [BECs]), inflammatory cells cellular debris in bronchi, intraepithelial neutrophils, alveolar emphysema, lesions (alveolar hemorrhage, significant alveolar edema, vasculitis/vascular endothelialitis), reactive inflammatory patterns (necrosuppurative bronchitis, intra-alveolar neutrophils, and macrophages, mononuclear infiltrate around airways, presence of polymorphonuclear granulocytes, perivascular mononuclear cuffs), as well as regeneration and repair (alveolar epithelial hyperplasia/regeneration). Masson’s trichrome (MT) staining was also performed to assess tissue architecture and gauge the progression of fibrosis. Inflammation and fibrosis were assessed by a blind observer. Images were acquired on Tissue Scope LE (Huron Digital Pathology, Waterloo, Canada) under the 20× objective. The region of aggregation of inflammatory cell infiltrates is delineated and represented as percentage of the cell infiltrates area to total tissue area. The regions of collagen content stained in blue were delineated and represented as percentage fibrosis area to total tissue area.

### Non-human primate challenge and analysis of viral loads

Three- to four-year-old male cynomolgus macaques were purchased from Worldwide Primates (Florida, USA). Animals were housed at the BSL2 facility at the University Health Network’s Animal Resources Centre (ARC) and were acclimatized for 30 days. Animals were vaccinated with 500 µL (1 × 10^8^ PFU) of either of the vaccine candidates (Fowl Adenovirus 9 expressing S-antigen from SARS-CoV-2 [FAdV9-S19] or Fowl Adenovirus 9 [FAdV-9]). During this time, animals were monitored for any adverse health effects and were bled weekly. After 28 days, animals from each group received 500 µL intranasal challenge dose of 1.78 × 10^6^ TCID50 of HCoV-229E. The virus was isolated from a mid-turbinate swab of a patient who underwent molecular respiratory multiplex testing and was only positive for HCoV-229E. Animals were monitored daily for symptoms. Blood was collected at various timepoints and was analyzed for cell counts and biochemistry using the VetScan VS2 chemistry analyzer and i-STAT analyzer (Zoetis, USA). Oral swabs and nasal washes were collected at multiple time points post-infection. At 28 days post-infection, animals were euthanized, and lungs, liver, kidneys, spleen, and heart were collected to evaluate pathology. Quantities of infectious virus were determined by adding liquid collected from oral swabs and nasal washes to Huh-7 cells. Huh-7 cells were seeded onto 96-well plates the previous day and were left overnight at 34°C. On the following day, media were aspirated, and samples were added and serially diluted (10-fold dilution) into subsequent wells, followed by 1 hour incubation at 34°C. After incubation, media were aspirated and replaced with complete DMEM, and cells were incubated at 34°C for 5 days. The TCID50 was determined using the Spearman-Karber method.

### Histopathology in non-human primates

Tissues were fixed in 10% neutral phosphate-buffered formalin, routinely processed, embedded in molting paraffin wax, sectioned at 5 μm, and stained with H&E for histopathologic examination. Masson’s trichrome staining was also performed to assess tissue architecture and gauge the progression of fibrosis. Images were acquired on Tissue Scope LE (Huron Digital Pathology, Waterloo, Canada) under the 20× objective. Tissue masking was set at 50% threshold—tiles with less than 50% MT stained by area will not be included, regardless of the H&E content. To quantify fibrosis and inflammation, an automated pipeline employing deep learning was created for the processing of Masson trichrome (MT) and H&E-stained image pairs. For each pair, the MT image was down-sampled by a factor of 16 and was registered to its H&E counterpart using the OpenCV python package. Next, the low-resolution MT image was color normalized to shift aniline blue, which stains collagen fibers, exclusively to the blue channel of the image. This channel was extracted, and the image was divided into 64 × 64 tiles (equivalent to 1,024 × 1,024 at the base resolution). A counting algorithm was used to assess blue pixel density and rank tiles to locate areas of abnormal collagen deposition. Reducing the resolution allowed a 10-fold increase in processing time while obtaining near-identical pixel density results when compared to the full-resolution image. The top 10% of tiles were used to reference their corresponding regions within the H&E images, which were accessed at full resolution for nuclear segmentation. To localize inflammatory nuclei, HoVer-Net(42), an H&E-specific instance segmentation model trained on the PanNuke Dataset, is used (42). This model simultaneously locates and classifies nuclei as “epithelial,” “connective,” and “inflammatory” (Fig. S3B). The density of inflammatory nuclei was calculated per tile as the ratio of inflammatory to epithelial and connective cells. Because only collagen-rich areas were analyzed, the pipeline assessed the colocalization of fibrosis (MT) and inflammation (H&E nuclei). The pipeline was written in Python 3.9 using OpenCV, Histolab, and TIAToolbox packages (42).

### Statistical analysis

All figures were generated using Prism version 9.2 (GraphPad Software Inc.), and statistical analysis was performed using *t*-tests and two-way analysis of variance (ANOVA) tests; *P* values of <0.05 were considered significant.

## Data Availability

Data will be made available upon reasonable request.

## SUPPLEMENTAL MATERIAL

Supplemental material jvi.01390-24-s0001.pdf Figures S1 to S4.

ASM does not own the copyrights to Supplemental Material that may be linked to, or accessed through, an article. The authors have granted ASM a non-exclusive, world-wide license to publish the Supplemental Material files. Please contact the corresponding author directly for reuse.
