# Supplementary material for: Vaccination against SARS-CoV-2 provides low-level cross-protection against common cold coronaviruses in mouse and non-human primate animal models
Source: J Virol. 2025 Jan 16;99(2):e01390-24. doi: 10.1128/jvi.01390-24 (PMC11853048; doi:10.1128/jvi.01390-24)

Supplemental Figure 1. Generation of recombinant vaccines. a) Verification of Fadmids by NotI digestion. M: 1kb DNA ladder. Lane 1: pFAdV-9-S19 (expected fragment sizes: 1391 bp, 6056 bp, 8834 bp, 11485 bp, 19730 bp). Lane 2: pFAdV-9wt (expected fragment sizes: 1391 bp, 6056 bp, 11485 bp, 26131 bp). **b)** Verification of recombinant viruses by PCR. M: 1 kb DNA ladder. Lane 1: rFAdV-9-S19 (expected fragment size: 5621 bp). Lane 2: wild-type FAdV-9 (expected fragment size: 3188 bp). **c)** Western blot analysis of whole cell lysates from CH-SAH cells infected with recombinant viruses to confirm S protein expression. M: Protein ladder. Lane 1: rFAdV-9-S19. Lane 2: FAdV-9.

Supplemental Figure 2. Blood biochemistry of NHPs following infection with HCoV-229E. Blood was collected from NHPs (n = 3 per group). before and after HCoV-229E challenge and amounts of **a)** Monocytes **b)** Neutrophils, **c)** Lymphocytes **d)** White blood cells (WBC), **e)** Total protein and **f)** creatinine were determined. Error bars represent standard deviation. Two-way ANOVA was used for statistical analysis. P value <0.001 (**), P value <0.05 (*).

Supplemental Figure 3. Schematics and sample images of our automated fibrosis (A-B) and inflammation quantification (C-D) pipeline for NHP lungs. (A) A whole-slide image (WSI) with Masson's Trichrome stain is first color-normalized, then the aniline blue stain is algorithmically extracted. The WSI is divided into 410 x 410-micron tiles, and a fibrosis score is calculated per tile based on blue pixel density. Tiles are ranked by their fibrosis scores, and the top 10% are selected for further analysis. **(B)** An example of a highly fibrotic tile with a dense band of aniline blue stain is shown. **(C)** Inflammation analysis is performed on a co-registered, H&E stained WSI. Matching tiles from **(A)** are extracted for inflammatory cell detection, and the ratio of inflammatory cells to all cells is calculated. **(D)** Inflammation analysis on an H&E tile

co-registered with the highly fibrotic tile shown in **(B)** reveals a high inflammatory cell ratio of 20%.

Supplemental Figure 4. HCoV-OC43 and HCoV-NL63 viral load in vaccinated and sham vaccinated mice. Viral loads were determined from oral swabs of hACE-2 K-18 mice challenged with **(A)** HCoV-OC43, and **(B)** HCoV-NL63. Error bars represent standard deviation. **(C)** Viral loads in lung tissues at 5-days post-infection with HCoV-OC43 or HCoV-NL63. Two-way ANOVA using Dunnett's multiple comparisons was used for analysis. P value <0.001 (**).

Supplemental Figure 1

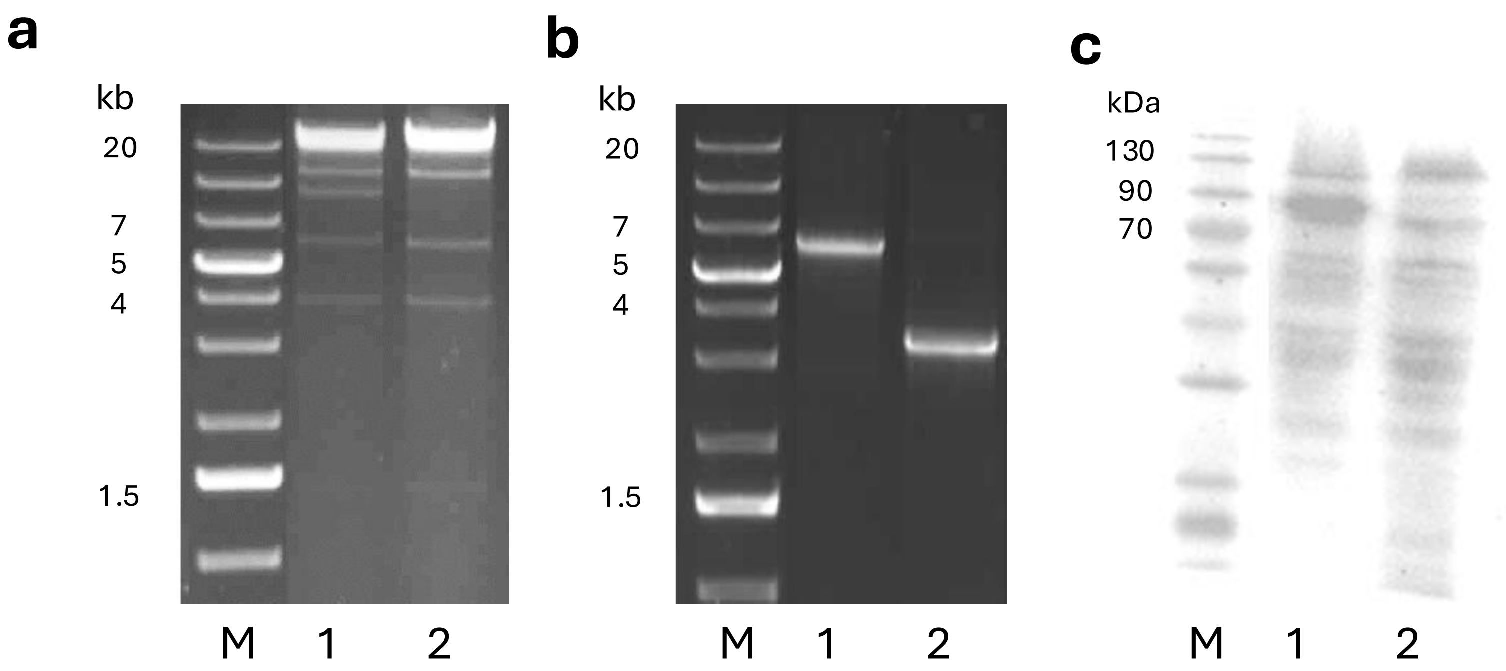

Supplemental Figure 2

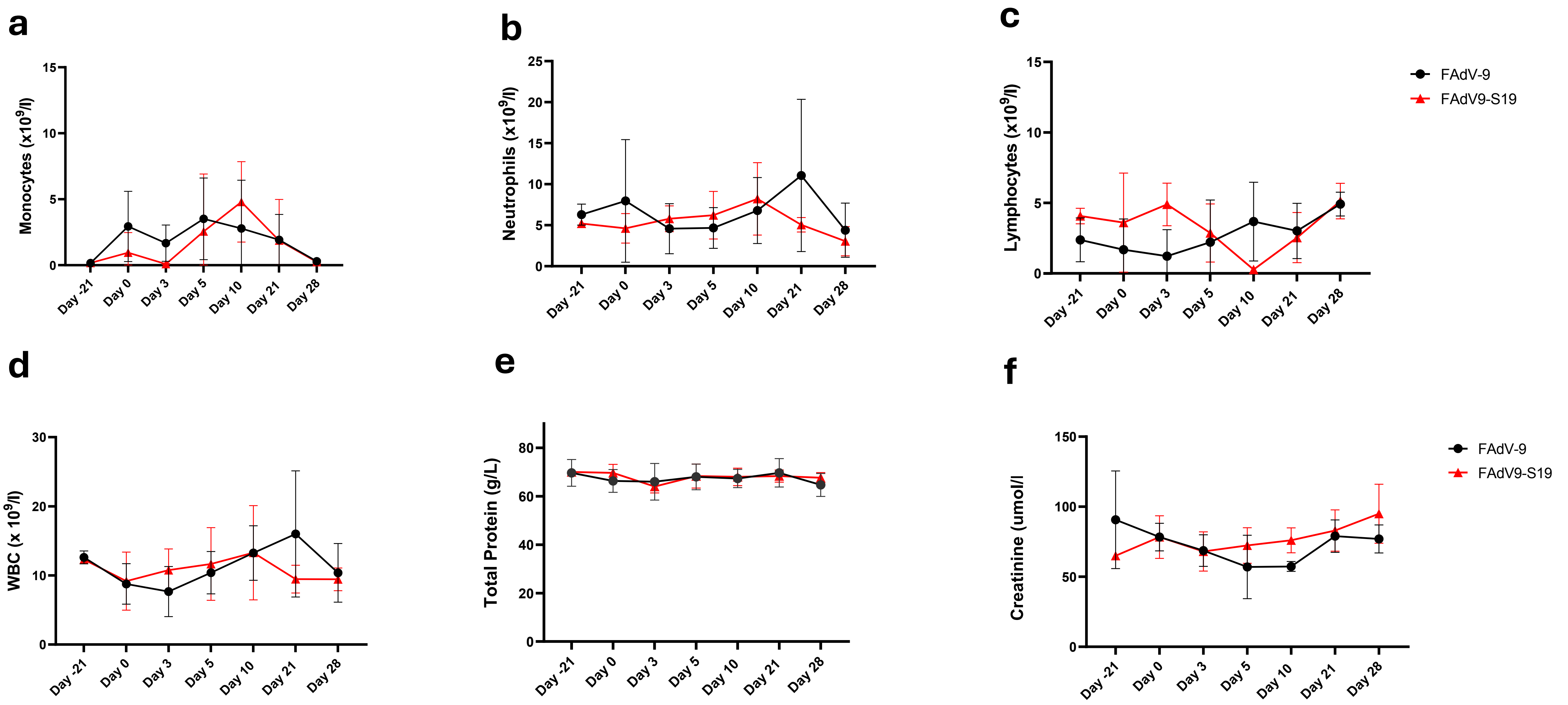

Supplemental Figure 3

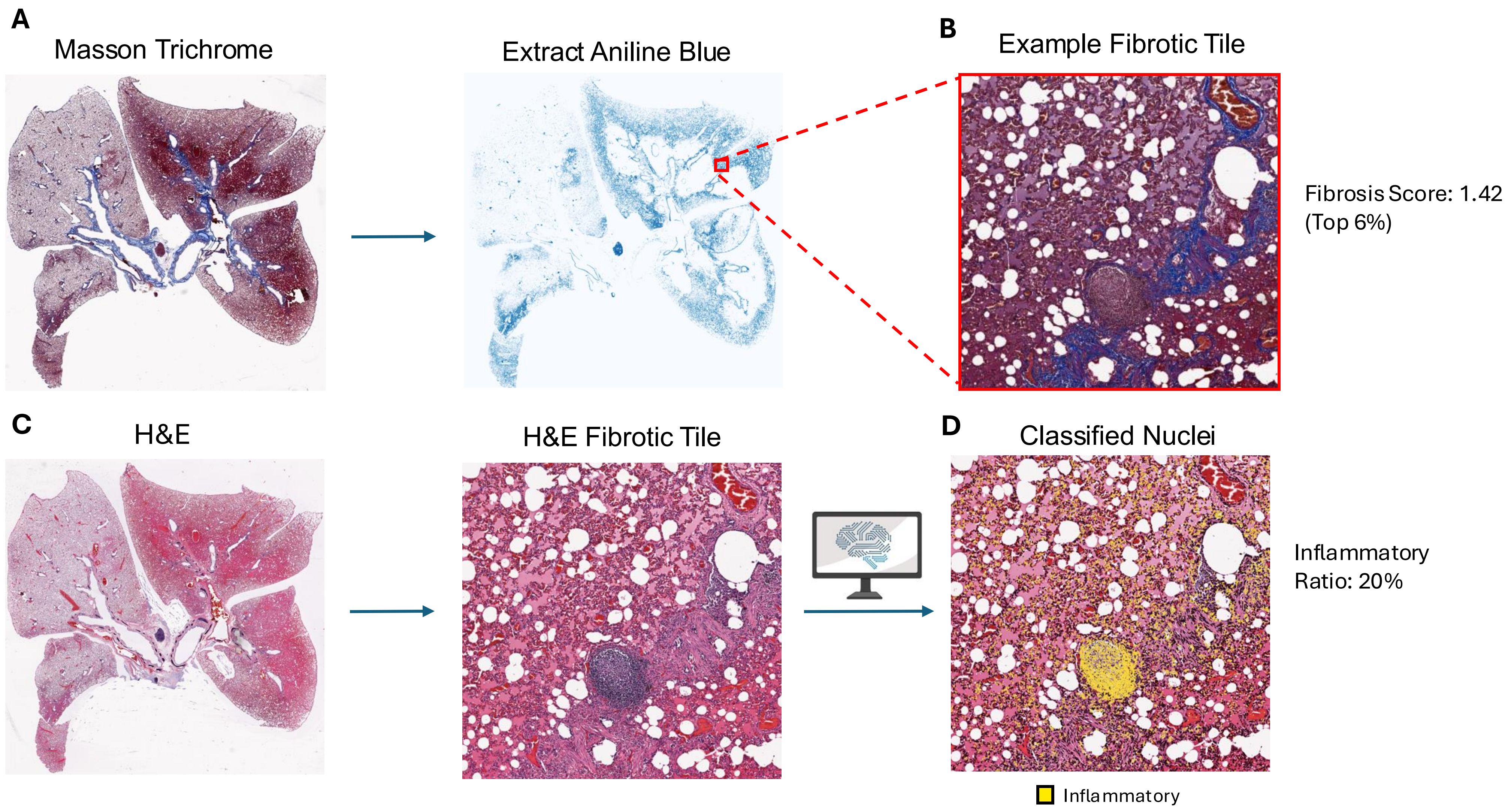

Supplemental Figure 4

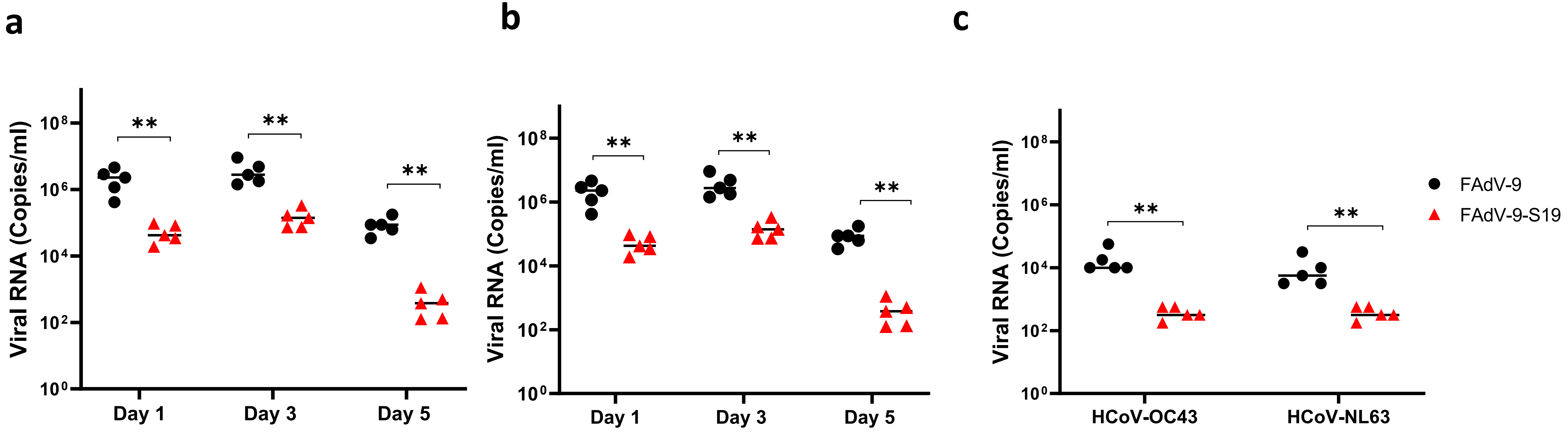

Supplement: Supplemental material — Figures S1 to S4. [file jvi.01390-24-s0001.pdf]
